# A Novel Role of Numb as A Regulator of Pro-inflammatory Cytokine Production in Macrophages in Response to Toll-like Receptor 4

**DOI:** 10.1038/srep12784

**Published:** 2015-08-05

**Authors:** Patipark Kueanjinda, Sittiruk Roytrakul, Tanapat Palaga

**Affiliations:** 1 Interdisciplinary Program in Medical Microbiology, Graduate School, Chulalongkorn University, Bangkok 10330, Thailand; 2 Center of Excellence in Immunology and Immune-mediated Diseases, Chulalongkorn University, Bangkok 10330, Thailand; 3 National Center for Genetic Engineering and Biotechnology (BIOTEC), National Science and Technology Development Agency, Pathum Thani, Thailand; 4 Department of Microbiology, and Omics Sciences and Bioinformatics Center Faculty of Science, Chulalongkorn University, Bangkok 10330, Thailand

## Abstract

Activation of macrophages triggers the release of pro-inflammatory cytokines leading to inflammation. Numb is a negative regulator of Notch signaling, but the role of Numb in macrophages is not fully understood. In this study, the role of Numb as a regulator of inflammatory responses in macrophages was investigated. Murine bone marrow-derived macrophages, in which expression of Numb was silenced, secreted significantly less TNFα, IL-6 and IL-12 and more IL-10 upon activation by lipopolysaccharide (LPS), a ligand for Toll-like receptor 4 (TLR4), despite increased Notch signaling. The *Tnf*α mRNA levels both in *Numb*-deficient and wild-type macrophages were not significantly different, unlike those of *Il6* and *Il12-p40*. In *Numb*-deficient macrophages, the *Tnf*α mRNAs were degraded at faster rate, compared to those in control macrophages. Activation of p38 MAPK and NF-κΒ p65 were compromised in activated *Numb* deficient macrophages. Numb was found to interact with the E3 ubiquitin ligase, Itch, which reportedly regulates p38 MAPK. In addition, blocking the Notch signaling pathway in activated, *Numb*-deficient macrophages did not further reduce TNFα levels, suggesting a Notch-independent role for Numb. A proteomics approach revealed a novel funciton for Numb in regulating complex signaling cascades downstream of TLRs, partially involving Akt/NF-κB p65/p38 MAPK in macrophages.

Macrophages are among the first immune cells to encounter microorganisms and initiate immune defense. Therefore, they play an important role in immune response. Macrophages can recognize and discriminate pathogens from self-molecules through binding of their surface and intracellular receptors to different exogenous and endogenous ligands. The receptors, known as pattern recognition receptors (PRR), can bind to many essential parts of pathogens, collectively called pathogen-associated molecular patterns (PAMPs). These interactions result in changes in the activation status of transcription factors such as MAPK and NF-κB that govern macrophage activation^1^,^2^, leading to production of various antimicrobial molecules such as anti-microbial peptides, cytokines and chemokines^3^. Unless these immune responses are tightly regulated, damage to host cells may occur.

One pathway that is crucial for the feedback inhibition of LPS signaling is the phosphoinisitide-3 kinase (PI-3K)-Akt/PKB signaling pathway. The Akt family of serine-threonine protein kinases is activated by PI-3K and plays a regulatory role in many cellular functions such as cell proliferation, differentiation, and metabolism. Stimulating macrophages with LPS activates PI-3K and its downstream target, Akt, which suppresses LPS-activated NF-κB and MAPK signaling pathways. This negative feedback loop results in decreased pro-inflammatory cytokine production^4^,^5^. All three isoforms of Akt (Akt1, Akt2, Akt3) have been implicated in modulating host immune defense in infection and autoimmunity^6^.

Evolutionarily conserved, Notch signaling has been reported to regulate development and function of cells in the immune system^7^,^8^. The Notch transmembrane receptors (Notch-1, -2, -3, and -4) are activated upon engagement of ligands (Jagged-1 and -2, Delta-1, -3, and -4). After ligand-receptor binding, the receptor undergoes enzymatic cleavage, generating a Notch intracellular domain (NICD) that can translocate to the nucleus. There it functions as a transcriptional co-activator through association with its DNA binding partner, CSL (CBF1, Suppressor of Hairless, LAG-1/RBP-Jκ) and other co-activators such as p300, to regulate transcription of specific target genes. Several target genes have been identified, including transcriptional repressors of the HES family (*Hes-1, Hes-2*)^9^.

Numb is a membrane-associated protein that contains an N-terminal phosphotyrosine-binding domain (PTB) and C-terminal proline-rich region (PRR), harboring putative Src homology 3-binding sites and Eps15 homology (EH) regions^10^. *In vitro* studies demonstrated that Numb interacted with various intracellular molecules of different signaling pathways, thereby regulating multiple cell functions. It has been extensively studied for its role in the maintenance of several types of neuronal stem cells during fetal development^11^,^12^ and in many types of cancers^13^,^14^,^15^. Wilson *et al.* used *Numb*-deficient mice to show that *Numb* was dispensable for the development of hematopoiesis and lymphopoiesis into myeloid and lymphoid cells, respectively^16^. Numb was also dispensable for T cell proliferation and function^17^. However, several studies demonstrated that Numb played critical roles in regulating asymmetric T cell division that occurred during development, differentiation, and in response to infection^18^,^19^.

The E3 ubiquitin ligase, Itch, a member of the HECT (homologous to the E6-AP C-terminus) family contains a C2 domain, four WW domains, and a HECT ligase domain^20^. These WW domains are implicated in protein-protein interactions, whereas the HECT domain helps in recruitment of E2 ubiquitin loading enzymes and transfers ubiquitin to their substrates^21^. Mice deficient in *Itch* developed a skin-scratching or “itchy” phenotype^22^ together with severe immune dysregulation, including lymphadenopathy, splenomegaly, and inflammation in the lungs and digestive tract^23^. In addition, Itch was shown to play a key role in peripheral T-cell tolerance. Disruption of Itch-mediated JunB ubiquitination results in continuous binding of JunB to *Il-4* promoter, thereby promoting a strong T_H_2 response^24^. Itch was also identified as a negative regulator of homeostasis and hematopoietic stem cell function by regulating the Notch signaling pathway^25^.

Numb can interact with Itch to antagonize Notch signaling via ubiquitin-mediated degradation of the Notch receptors^26^,^27^, raising the possibility that Numb may play a critical role in other types of immune cells in which Notch signaling is involved. Studies from our group and others suggest that, upon stimulation of macrophages, Notch signaling is activated and this activation up-regulates gene expression patterns involved in macrophage activation, including *Irf1*, *Socs1*, and *H-2A*, as well as the production of both pro- and anti-inflammatory cytokines such as TNFα, IL-6, IL-12, and IL-10^27^,^28^,^29^. These studies demonstrated that using γ-secretase inhibitors or *Notch1* RNAi to interfere with Notch signaling dampened macrophage activation and abrogated pro-inflammatory cytokine production. In this study, we describe a novel role for Numb in positively regulating TNFα, IL-6, and IL-12 cytokine production. Furthermore, Numb interacts with Itch which, in turn, regulates downstream signaling pathways, including NF-κB p65 and p38 MAPK. Moreover, we utilized a quantitative proteomics approach, combined with analysis of a bioinformatics database to propose that the Numb/Akt1 axis regulates pro-inflammatory cytokines in macrophages.

## Materials and Methods

### Construction of retrovirus-based shRNA plasmids and generating retrovirus particles for transduction

To construct pMKO.1-*shNumb*-GFP plasmid, the pMKO.1-GFP retroviral vector was used as a backbone vector (Addgene plasmid #10676, USA). Small hairpin loop-forming double-stranded oligonucleotides containing a *Numb*-specific sequence (5′- AACCACTTTCACAAGAGAAGG -3′)^30^, which targets the murine *Numb* gene, was inserted into pMKO.1-GFP. To obtain retroviral particles containing the plasmids, human embryonic kidney 293T cells (ATCC CRL-3216) were cultured in DMEM supplemented with 10% FBS at a concentration of 4 × 10^5^ cells/ml for 24 hrs and transfected with pMKO.1-*shNumb*-GFP or pMKO.1-GFP, as a control vector. Cells were maintained in DMEM media supplemented with 10% FBS in 5% CO_2_ incubator at 37 °C. Two days later, the cell culture supernatants were collected and filtered through 0.45 μm filter and used as retroviral particles for transduction.

### Generation of bone marrow-derived macrophages (BMMs) and transduction by retroviral plasmid

C57BL/6 mice were purchased from the National Laboratory Animal Center (Mahidol University, Salaya, Thailand). All procedures involving laboratory animals were reviewed and approved by Chulalongkorn University Institutional Animal Care and Use Committee (CU-IACC protocol review number #001/2555-20/54). All the procedures involving laboratory animals were carried out in accordance with the guidelines issued by CU-IACC. Bone marrow cells were harvested and filtered through 70 μm nylon mesh. The cells were incubated in L929-conditioned medium at a density of 6.25 × 10^5^ cells/mL and maintained in a 5% CO_2_ incubator at 37 °C. Two days later, cells were harvested and seeded at 1 × 10^6^ cells/well in 12-well tissue culture plates. The supernatant from 293T cell culture containing retroviral particles was mixed with X-tremeGENE HP transfection reagent (Roche, Germany) and added to the BM culture. After 1 hr of incubation in a 5% CO_2_ incubator at 37 °C, L929-conditioned medium was added to the BM cultures and incubation continued for 24 hrs. The transduction steps were repeated using freshly prepared retroviral partcles. L929-conditioned medium was added to the BM cultures every 3 days during 7-day incubation period. On day 8 after the first transduction, cells were harvested for cell sorting using FACSAria II (BD Biosciences) to obtain GFP^+^ cells. The sorted cells were seeded at 5 × 10^4^ cells/well in 24-well plates and incubated for 5 days. The cells were harvested and the purity of F4/80^+^ GFP^+^ cells was >90%, as determined by flow cytometry.

### Overexpression of Numb in macrophage-like cell line RAW264.7

RAW264.7 cells (ATCC TIB-71) were transfected with either pCI-OVA^31^, as a control, or pCIneoHA mouse Numb^65^(Addgene plasmid #37012) using FuGENE HD transfection reagent (Promega, USA). After transfection for 24 hrs, RAW264.7 cells were stimulated with 100 ng/mL *E. coli* LPS (Sigma Aldrich, USA) and protein lysates or culture supernatants were collected for further analysis.

### Reagents

BMMs were activated by 100 ng/mL of *Escherichia coli* lipopolysaccharide (LPS) (Sigma-Aldrich, USA). The γ-secretase inhibitor (GSI), DAPT (Merck, Germany), was used to inhibit Notch signaling as previously described^27^,^29^. To inhibit Notch signaling in BMMs, 25 μM of DAPT or DMSO, as vehicle control, was added to the cells for 1 hr prior to stimulation with LPS.

### mRNA decay assay

To assess mRNA stability, cells were seeded at 1 × 10^4^ cells/well in 96-well plates and maintained in DMEM supplemented with 10% FBS in 5% CO_2_ incubator at 37 °C for 24 hrs. The cells were pre-treated with LPS for 1 hr prior to treatment with actinomycin D (Merck, Germany) at the final concentration of 20 μg/mL for indicated time periods. Total RNA was isolated from the cultured cells with TRIzol reagent (Invitrogen, UK) as described in the manufacturer’s instructions and reverse transcribed to cDNA for quantitative real-time PCR analysis.

### Co-immunoprecipitation

Briefly, 5 × 10^5^ BMM cells were maintained in complete DMEM supplemented with 10% FBS. Cells were stimulated with 100 ng/ml *E. coli*-derived LPS for 30 mins. After stimulating, cells were washed and detached using cold PBS. Cell pellets were lysed by vigorous vortexing in 0.5% NP-40 lysis buffer containing protease inhibitors and phosphatase inhibitor (Roche, Germany) and lysis was allowed to continue on ice for 30 minutes. Cells were centrifuged at 4 °C and protein lysates were transferred to new tubes. Protein concentration of each sample was determined using a BCA Assay Protein Assay kit (Pierce, USA). Protein A agarose beads (Cell Signaling Technology, USA) were used for the pre-clearing step of the immunoprecipitation. For immunoprecipitation, 250 ng of anti-IgG or anti-Numb antibody (Cell Signaling Technology, USA) was added to cell lysates and rotated on a rocking platform overnight.

### Immunoblotting

After stimulation with LPS at indicated time points, whole cell lysates were extracted using RIPA lysis buffer containing protease inhibitor cocktail and a phosphatase inhibitor (Roche, Germany). Protein concentrations were measured using a BCA Protein Assay kit (Pierce, USA) following the manufacturer’s instructions. Antibodies used in this study were anti-Notch1 (clone C-20), anti-GAPDH (Santa Cruz Biotechnology, USA), anti-ITCH (Epitomics, USA), anti-cleaved Notch1 (Val1744), anti-Numb, anti-phospho NF-κB p65, anti-NF-κB p65, anti-phospho-p38 MAPK, anti-p38 MAPK, anti-phospho-p42/44 MAPK, anti-p42/44 MAPK, anti-phospho-SAP/JNK MAPK, anti-SAP/JNK MAPK, anti-pAkt (Thr308), anti-Akt, HRP-conjugated anti-rabbit IgG (Cell Signaling Technology, USA).

### Semi-quantitative RT-PCR (qPCR)

Total RNA was isolated from cultured cells with TRIzol reagent (Invitrogen, UK) as described in the manufacturer’s instructions, and was reverse transcribed to cDNA as described previously^28^. Primers specific for *Numb* were designed (forward 5′-ACTACGGCAAAGCTTCAGGA-3′, reverse 5′-TGCATTCCTCTTGACTCATCA-3′), *Numb-like* (forward 5′-TACGGTTGAATGAGCTGCCA-3′; reverse 5′-AGGCAGAAGTCCCTG TTGTG-3′) and primers for *Hes1, Tnfα, IL-6, IL-12p40, IL-10, Ifn-*β*, Akt1, Akt2, Ticam1, Map3k10,* and *Gapdh* were used as previously described^28^,^29^,^32^,^33^,^34^,^35^. Semi-quantitative real-time PCR was carried out using MJ Mini Personal Thermal Cycler and iQ SYBR Green system (Bio-Rad, USA) following the manufacturer’s instructions. The relative gene expression levels were analyzed by CFX Manager (Bio-Rad, USA) and calculated as described previously^36^.

### ELISA

Cell culture supernatants were collected at indicated times after treatment with specific inhibitor and stimulation with LPS. To assess protein levels of TNFα, IL-6, IL-10, and IL-12, ELISA were performed using LEGEND MAX^TM^ mouse TNFα, IL-6, IL-10, and IL-12p40 ELISA kits (BioLegend, USA) according to the manufacturer’s instructions.

### Flow cytometry

For intracellular cytokine staining, cells were treated with 1 μL of GolgiPlug^TM^ (BD Biosciences, USA) according to the manufacturer’s instructions, just before stimulation with LPS. BD Cytofix/Cytoperm^TM^ kit (BD Biosciences, USA) was used for staining according to the manufacturer’s instructions. At indicated time points, cells were harvested, washed, and incubated with 2.4G2 antibody (BD Biosciences, USA) to block FcγRII/III receptors. Antibodies used in this study: PE-labeled anti-F4/80, biotin-conjugated anti-CD11b, avidin-conjugated PECy5 (BioLegend, USA), PE-labeled anti-MHC class II, biotin-conjugated anti-CD86 (BioLegend, USA), avidin-conjugated ECD (Beckman Coulter, USA), PE-labeled anti-TNFα, PE-labeled anti-IL-6, and biotin-conjugated IL-12p40 and avidin-conjugated PECy5 (BioLegend, USA). The acquired data were analyzed using FlowJo vX (TreeStar, USA).

### Protein preparation and proteomics data analysis

BMM containing shRNA plasmids were prepared as previously described. The cells were seeded overnight at 2.5 × 10^5^ cells/well in 12-well plates in L929-conditioned medium. On the next day, cells were stimulated with 100 ng/ml of *E. coli* LPS (Sigma-Aldrich, USA) for 30 mins and were harvested using RIPA lysis buffer. Cell lysates were stored at −80 °C until use. Protein concentration of the samples were measured by Lowry method^37^. The absorbance at 750 nm (OD_750_) was measured and protein concentration was calculated using the bovine serum albumin standard curve. Protein separation by SDS-PAGE, in-gel digestion, and proteomic data analysis were processed as described in Supplementary Materials.

### Statistical analysis

All experimental data in this study were presented as the means ± SEM of three independent experiments or from a representative experiment of three independent experiments unless indicated otherwise. The statistical significances of the differences in the experimental data were evaluated using the Student’s *t* test. A *P* value of < 0.05 was considered to be significant.

## Results

### Numb expression is decreased in LPS-stimulated macrophages and silencing *Numb* increases activation of Notch signaling

**Figure 1:**
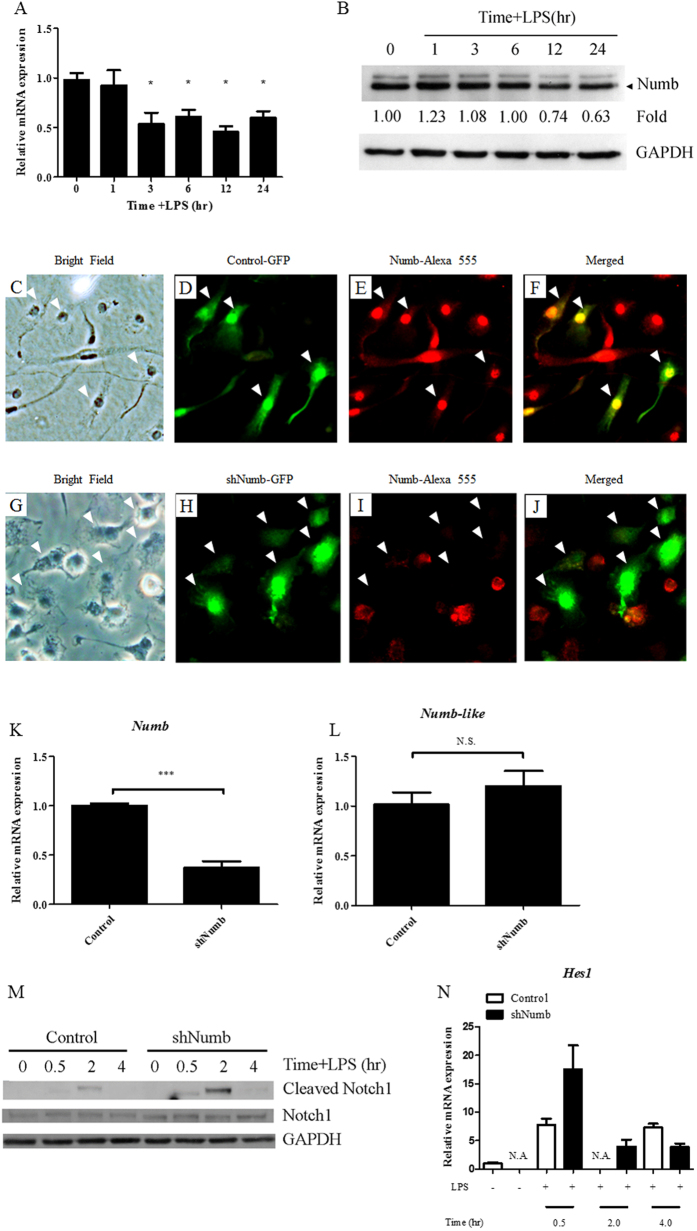
Numb expression in macrophages after LPS stimulation and effects of *Numb* silencing on Notch signaling. (**A**) Bone marrow-derived macrophages were stimulated with LPS (100 ng/mL) and *Numb* mRNA was measured. (**B**) Numb protein expression was shown and its intensity was measured as fold-increase compared to unstimulated cells. (**C**–**J**) Immunofluorescent staining of macrophages retrovirally infected with control vector (**C**–**F**) or sh*Numb* vector (**G**–**J**), and stained for Numb (red). White arrows indicate macrophages containing shRNA vector (green). (**K**–**L**) Expression level of *Numb* (**K**) and *Numb-like* (**L**) mRNA from GFP^+^ macrophages as determined by qPCR. (**M**) Cleaved Notch1 (Val1744) was detected in LPS-stimulated macrophages by immunoblot. (**N**) *Hes1* mRNA was measured in LPS-stimulated macrophages by qPCR. N.D. = not detectable. Data are the mean ± SEM from three independent experiments, and a representative blot from at least two independent replicates.

We began our investigation by exploring the expression of Numb in LPS-stimulated macrophages. We found that the expression level of *Numb* mRNA significantly decreased to approximately one-half and remained at this level after LPS stimulation for 3 hrs and up to 24 hrs (Fig. 1A). For protein expression, we observed a slight increase of Numb at 1 hr post stimulation and a gradual decrease over the next 24 hrs (Fig. 1B and Supplementary Figure 1A). To further investigate the effect of Numb in macrophages, we constructed a retroviral shRNA vector, pMKO.1-*shNumb*-GFP, introduced it into BMMs and confirmed its target specificity. In BMMs retrovirally transduced with pMKO.1-*shNumb*-GFP vectors, the expression level of Numb decreased significantly compared to that of the control-transduced macrophages (Fig. 1C–J). To further assess the target specificity of the construct, macrophages containing pMKO.1-*shNumb*-GFP, or control vector, were sorted to obtain homogenous GFP^+^ populations. The mRNA level of *Numb* and its homolog, *Numb-like*, that has been reported to have redundant functions with Numb in other cell types were measured^30^,^38^,^39^. The expression level of *Numb* mRNA was reduced to one-half, whereas the level of *Numb-like* mRNA was not affected (Fig. 1K,L). These results confirmed that the shRNA vector specifically targeted only *Numb*. Numb has been reported to be a negative regulator of Notch signaling. When we assessed cleaved Notch1 expression (Val1744), and the level of *Hes1* mRNA, we observed that both were increased in LPS-activated *Numb*-silenced macrophages, compared to control cells (Fig. 1M,N). These results confirmed the role of Numb as a negative regulator of Notch signaling, as previously described.

### Numb is dispensable for differentiation of bone marrow macrophages *in vitro*

**Figure 2:**
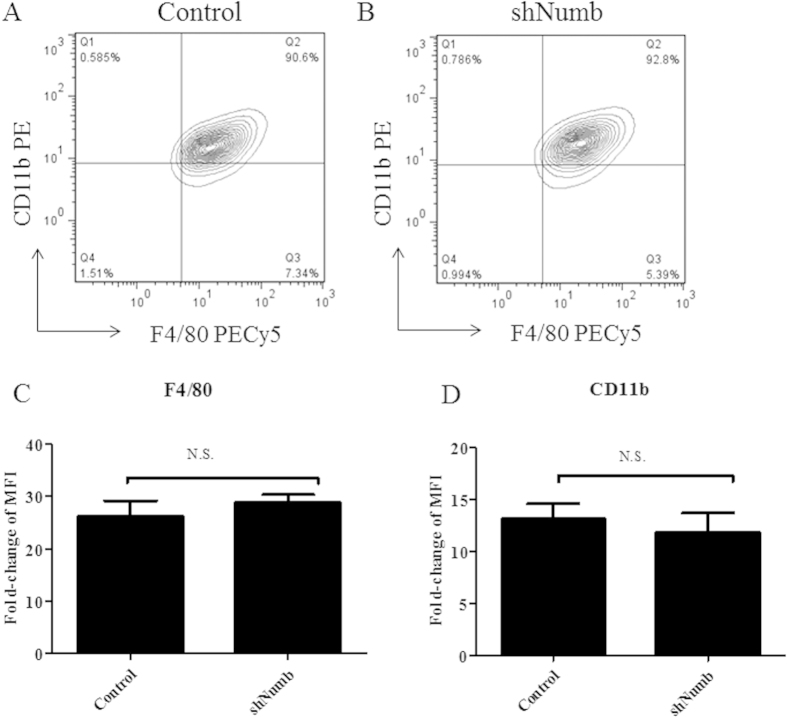
Differentiation of bone marrow cells to macrophages upon silencing of Numb. (**A**,**B**) Expression of macrophage markers, CD11b and F4/80, from GFP^+^ population of macrophages that were retrovirally infected with control (**A**) or sh*Numb* (**B**) vectors was determined by flow cytometry. (**C**,**D**) Expression level of F4/80 (**C**) and CD11b (**D**) from (**A**) and (**B**) were shown as fold-change of mean fluorescence intensity (MFI). Data are the mean ± SEM from three independent experiments. N.S. = not statistically significant.

A report by Wilson *et al.*^16^ demonstrated that hematopoietic stem cell compartments, including macrophages, developed normally in *Numb* and *Numb-like* double-knockout mice. Our system employed an insertion of the retroviral vector in hematopoietic cells from the bone marrow, causing constitutive silencing of *Numb*. We, therefore, tested whether silencing of *Numb* affects the development of macrophages from bone marrow hematopoietic stem cells *in vitro*. After retroviral transduction of pMKO.1-*shNumb*-GFP or control vectors, bone marrow cells were induced to differentiate to macrophages. Cells containing the vectors were gated for GFP^+^ and assayed for the expression of two macrophage-specific surface markers, F4/80 and CD11b. Our results showed that the expression levels both of F4/80 and CD11b were comparable between control and *Numb*-silenced macrophages (Fig. 2A–D), indicating that *Numb* was dispensable for macrophage differentiation *in vitro*.

### *Numb*-deficient macrophages secreted less pro-inflammatory cytokines

**Figure 3:**
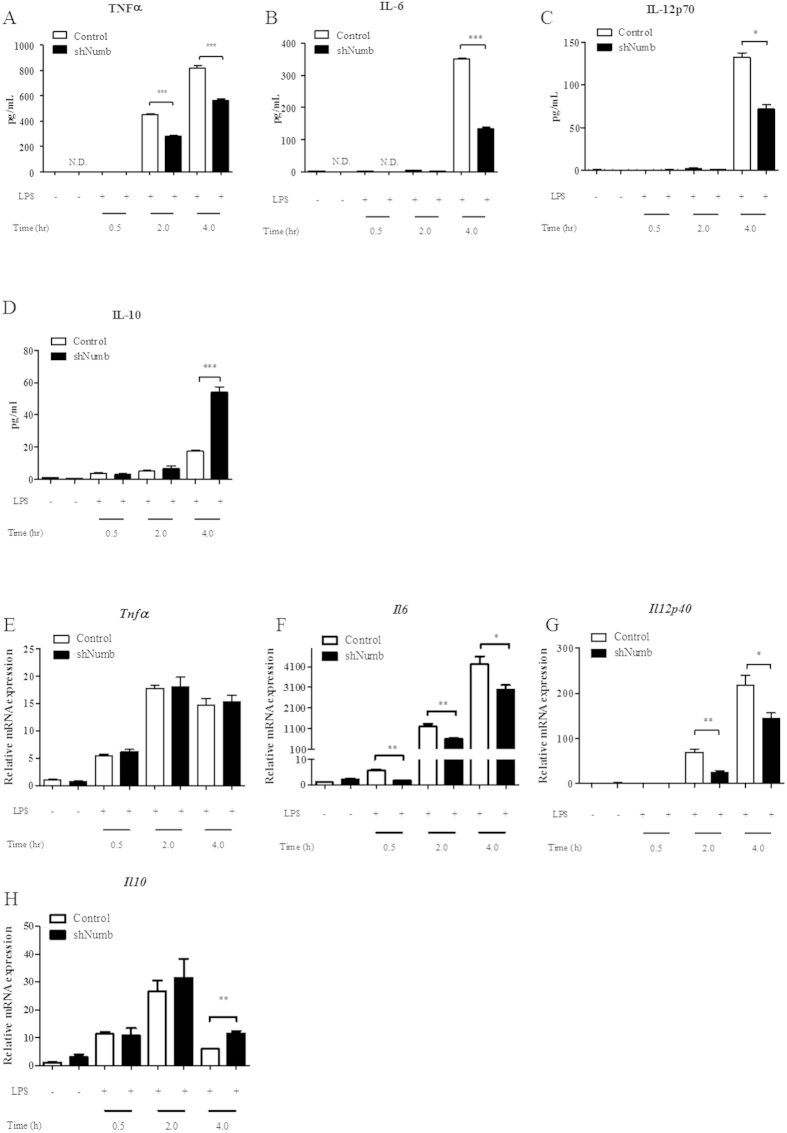
Expression of cytokines in macrophages lacking Numb after LPS stimulation. (**A**–**D**) Secretion levels of TNFα (**A**), IL-6 (**B**), IL-12p70 (**C**), and IL-10 (**D**) from control- (open bars) or sh*Numb*-infected macrophages (closed bars) following LPS stimulation were measured by ELISA. (**E**–**H**) mRNA levels of *Tnfα* (**E**), *Il6* (**F**), *Il12p40* (**G**), and *Il10* (**H**) from control- or sh*Numb*-infected macrophages following LPS stimulation were measured by qPCR. Data are the mean ± SEM from the representative experiment of two independent experiments performed in triplicates.

Our group and others reported that abrogating Notch signaling diminishes the ability of macrophages to secrete pro-inflammatory cytokines and perform other effector functions^27^,^28^,^29^,^40^. Therefore, we hypothesized that pro-inflammatory cytokines under the regulation of Notch signaling would increase in macrophages wherein *Numb* was silenced. Surprisingly, we found that *Numb*-silenced macrophages secreted significantly less TNFα, IL-6, and IL-12 after LPS stimulation (Fig. 3A–C). In contrast, the anti-inflammatory cytokine, IL-10, was higher in *Numb*-silenced macrophages (Fig. 3D). Furthermore, the expression of the costimulatory molecule CD86 decreased significantly (Supplementary Figure 1B) after LPS stimulation, whereas the expression of MHCII was not different between LPS-activated *Numb*-sufficient and *Numb-*deficient macrophages (Supplementary Figure 1C). These results suggest that Numb positively regulates pro-inflammatory programs in macrophages. We next investigated cytokine mRNA expression. We observed no difference in the levels of *Tnfα* between *Numb*-deficient and control macrophages (Fig. 3E). In contrast, the levels of *Il6* and *Il12p40* were readily down-regulated in *Numb*-deficient macrophages at all time points tested (Fig. 3F,G). In addition, the mRNA level of *Il10* and *Ifn*β was increased at 4 hrs after stimulation (Fig. 3H and data not shown). From these results, we conclude that the expression of *Il6, Il12p40* and *Il10*, but not *Tnfα*, are regulated by Numb at the transcriptional level.

### Numb regulates production of TNFα in a Notch-independent manner

**Figure 4:**
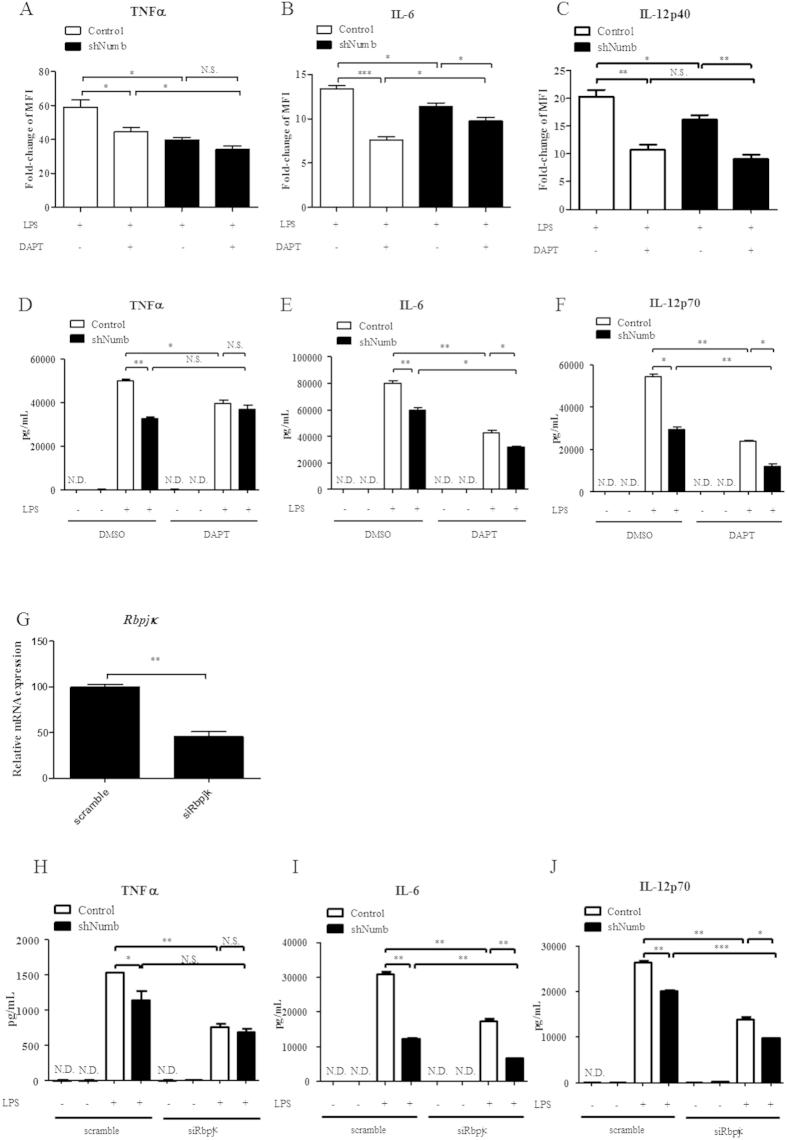
Expression of pro-inflammatory cytokines in macrophages lacking both Numb and Notch signaling after LPS stimulation. (**A**–**F**) GFP^+^ macrophages containing control (open bars) or sh*Numb* (closed bars) vectors were pretreated with DAPT for 1 hr prior to LPS stimulation. The levels of intracellular TNFα, IL-6, and IL-12 were determined by flow cytometry (**A**–**C**) or ELISA (**D**–**F**). N.D. = not detectable. (**G**) Expression level of *Rbpj*κ from macrophages transduced with si*Rbpj*κ, or scrambled siRNA as control, was measured by qPCR. (**H**–**J**) Expression levels of TNFα, IL-6, and IL-12p70 from GFP^+^ macrophages containing control (open bars) or sh*Numb* (closed bars) vectors which were transfected with scramble siRNA or si*Rbpjk* were measured by ELISA. N.D. = not detectable.

Our earlier observation revealed that Notch signaling was up-regulated in *Numb*-deficient macrophages following LPS stimulation. Previous reports have also demonstrated that Notch regulates pro-inflammatory cytokines in macrophages, especially IL-6 and IL-12p40^27^,^28^,^32^,^40^. Therefore, to elucidate the regulatory mechanism downstream of Numb, we used a γ-secretase inhibitor, DAPT, to pharmacologically inhibit Notch signaling in macrophages prior to and during LPS stimulation. As expected, intracellular and secreted TNFα, IL-6, and IL-12p40 protein level in DAPT-treated, *Numb*-intact macrophages decreased after LPS stimulation (Fig. 4A–F). However, there was not a significant difference in TNFα levels in *Numb*-silenced macrophages treated with DMSO or DAPT (Fig. 4A,D). In contrast, the levels of intracellular and secreted IL-6 (Fig. 4B,E) and IL-12p40 (Fig. 4C,F) in DAPT-treated, *Numb*-deficient macrophages were lower than those of the DMSO-treated, *Numb*-deficient cells. These results suggest that Notch and Numb counter-regulate expression of IL-6 and IL-12p40, but not TNFα. To confirm this, we silenced *CSL/RBP-J*κ, which plays a central role in canonical Notch signaling^41^. The mRNA level of *CSL*/*Rbp-j*κ was reduced by ~70% (Fig. 4G). Consistent with the results obtained by the treatment with DAPT, the levels of IL-6 and IL-12p40, but not of TNFα, in *Numb*-deficient macrophages were further decreased, compared to the those in macrophages transfected with scramble siRNA (Fig. 4H–J). Despite increased activation of Notch signaling in *Numb*-deficient macrophages, a reduction in TNFα expression that results from silencing *Numb*, demonstrates TNFα is not under the direct influence of Notch signaling. By contrast, it appears IL-6 and IL-12p40 expression are, at least partially, directly regulated by canonical Notch signaling.

### Activation of the NF-κB and p38 MAPK pathways are affected by decreasing Numb

**Figure 5:**
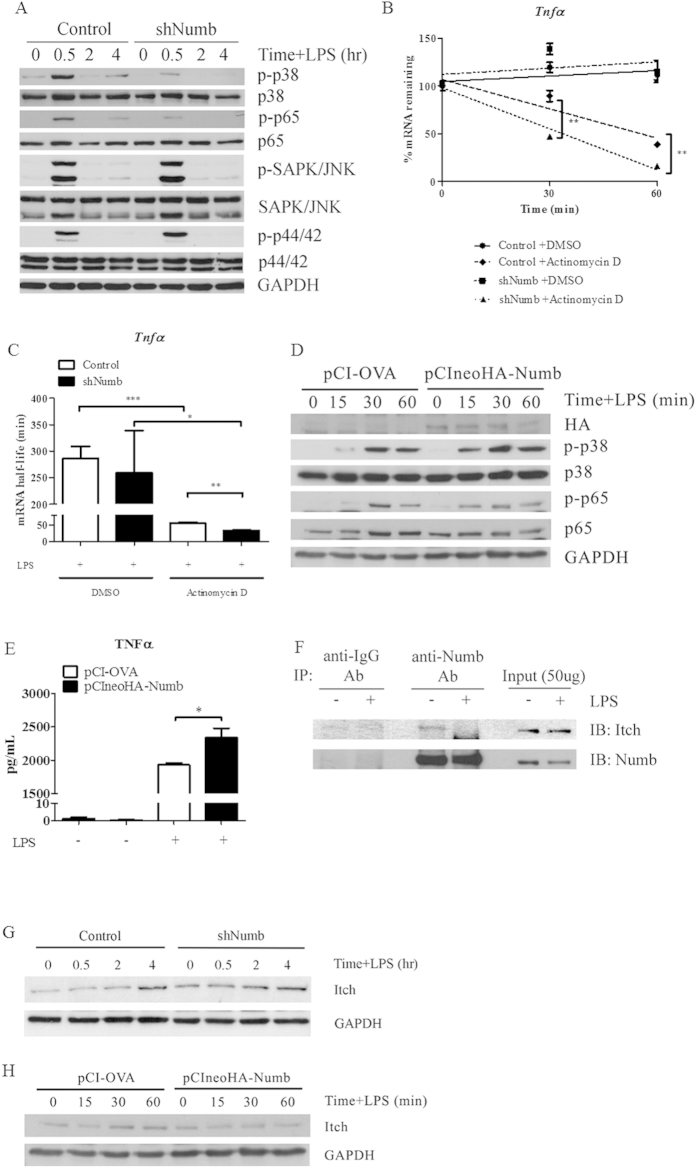
Effect of silencing *Numb* on activation of MAPK and NF-κB pathways in activated macrophages and the interaction of Numb and Itch. (**A**) After stimulation with LPS for indicated times, phosphorylation levels of NF-κB p65, p38, ERK1/2, and JNK MAPK from GFP^+^ macrophages were analyzed. Data are representative of two independent experiments. (**B**,**C**) GFP^+^ macrophages containing control (open bars) or sh*Numb* (closed bars) vectors were stimulated with LPS for 1 hr prior to treatment with DMSO or with actinomycin D to inhibit mRNA synthesis. The relative amount of remaining *Tnfα* mRNA was measured by qPCR. Half-life of *Tnfα* mRNA was calculated using linear regression line equations and shown in (**C**). Data are the mean ± SEM from representative of two independent experiments performed in triplicates. (**D**) RAW264.7 cell line was transiently transfected with the control plasmid or pCIneoHA-Numb and stimulated with LPS as indicated. p38 MAPK and NF-κB p65 were detected by immunoblotting. (**E**) RAW264.7 cell line transfected with the control plasmid or pCIneoHA-Numb were stimulated with LPS for 1 hr. The amount of TNFα was measured by ELISA. (**F**) Co-immunoprecipitation of endogenous Numb from unstimulated or LPS-stimulated macrophages was analyzed by immunoblotting. (**G**) Expression of Itch in macrophages containing control or sh*Numb* vector was detected by immunoblotting following LPS stimulation. (**H**) RAW264.7 cell line transfected with the control plasmid or pCIneoHA-Numb were stimulated with LPS for the times indicated and the level of Itch was detected by immunoblotting.

Regulatory mechanisms of TNFα, IL-6, and IL-12p40 expression have been extensively studied and shown to be regulated through different MAPK and NF-κB pathways^1^,^2^,^42^. Therefore, we tested the effect of *Numb* silencing on activation of MAPK and NF-κB pathways in macrophages. We found that the phosphorylation levels of both NF-κB p65 (RelA) and p38 MAPK were compromised in *Numb*-deficient macrophages after LPS stimulation, while the phosphorylation levels of ERK1/2 and SAP/JNK MAPKs remained similar between control and *Numb*-deficient macrophages (Fig. 5A). This selective effect silencing *Numb* has on p38 MAPK and NF-κB p65 activation following TLR4 stimulation may act to decrease TNFα, IL-6 and IL-12p40. Our previous results showed that the mRNA levels of *Tnfα* did not differ between *Numb*-deficient and control macrophages, consistent with several studies that demonstrated *Tnfα* mRNA is regulated by p38 MAPK, followed by post-transcriptional regulation^43^,^44^. Therefore, to address whether Numb exerts an effect on *Tnf*α mRNA stability, we performed a *Tnfα* mRNA decay assay, using actinomycin D to inhibit mRNA synthesis. We found that *Tnfα* mRNA was degraded at a faster rate in activated macrophages wherein *Numb* was silenced (Fig. 5B,C). On the other hand, we found that the mRNA stability of *Il6* was similar in the presence or absence of Numb (Supplementary Figure 2). Taken together, these results suggested that Numb positively regulates phosphorylation of p65 in the NF-κB pathway, possibly facilitating *Il6* and *Il12p40* mRNA transcription, as well as phosphorylation of p38 in MAPK, which may further mediate post-transcriptional regulation of *Tnfα* mRNA.

To complement the silencing approach, an overexpression of Numb in macrophage-like cell line, RAW264.7 was performed. As shown in Fig. 5D, Numb overexpression resulted in increased activation of p38 MAPK and NF-κB p65. Furthermore, increased TNFα production was detected, as expected if Numb functions to regulate TNFα (Fig. 5E).

Numb was reported to interact with Itch to promote ubiquitin-mediated degradation of Notch receptors^45^, and a recent report demonstrated that Itch functioned as a negative regulator of p38α^46^. Therefore, we hypothesized that Numb may regulate the activation of p38 MAPK through Itch. To test our hypothesis, we immunoprecipitated Numb and determined its physical interaction with Itch, using immunoblotting techniques. Our result show that Numb and Itch could be found in a complex in resting macrophages, but Itch dissociated from Numb upon LPS stimulation (Fig. 5F). Furthermore, Itch expression was higher in *Numb*-silenced macrophages, compared to those in control macrophages and, in contrast, Numb overexpression reduced Itch level (Fig. 5G,H). These results suggested that Numb and Itch interact physically and may affect activation of p38 MAPK and NF-κB p65.

### Proteomics analysis identifies Akt1 as a potential regulator of pro-inflammatory cytokines in *Numb*-deficient macrophages

**Figure 6:**
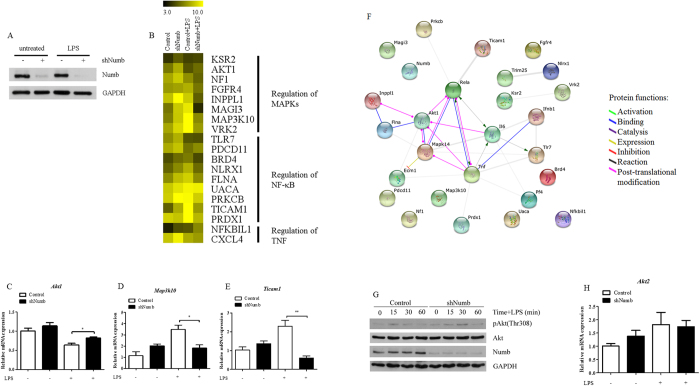
Proteomic data analysis of proteins from *Numb*-silenced macrophages. (**A**) Expression level of Numb in GFP^+^ macrophages following LPS stimulation was detected by immunoblotting. (**B**) Heat map representing expression levels of proteins as identified by GO enrichment in biological process. (**C**–**E**) Expression level of *Akt1* (**C**), *Map3k10* (**D**), and *Ticam1* (**E**) in control (open bars) or sh*Numb* (closed bars) macrophages were detected by qPCR. (**F**) A network of protein-protein interactions generated from proteins in (**B**) using the software STRING v9.1. (**G**) Phosphorylation of Akt (Thr308) and total Akt from macrophages containing control or sh*Numb* vector was detected by immunoblotting. (**H**) Schematic diagram showing how Numb interacts with different proteins and regulates pro-inflammatory cytokines (see text for details). Solid lines depict the confirmed links while the dotted lines depict the potential links.

To investigate further the role of Numb in macrophages, we employed a quantitative proteomics approach using *Numb*-silenced macrophages, or control cells, stimulated with LPS. The expression of Numb was separately confirmed (Fig. 6A) prior to gel loading for SDS-PAGE followed by trypsin digestion. Based on the LC/MS/MS data, we identified 758 proteins that were differentially expressed in the unstimulated or LPS-activated *Numb*-intact and *Numb*-deficient macrophages (Supplementary Figure 3). Among these proteins, 551 had previously described functions and so were subjected to analysis for protein-protein interaction using the software STRING v9.1^47^. Gene ontology (GO) enrichments in biological processes of these proteins using the software identified categories of proteins involved in regulating MAPKs, NF-κB signaling pathways and TNF production as represented by a heat map (Fig. 6B). To validate our proteomics results, we performed qPCR to measure *Akt1*, *Map3k10*, and *Ticam1* in *Numb*-silenced macrophages and found that their expression pattern correlated well with our proteomics data (Fig. 6C–E).

To further predict whether these proteins were involved in the regulation of pro-inflammatory cytokine production in *Numb*-deficient macrophages, TNFα, IL-6, IFN-β, Numb, NF-κB p65 (RelA), and p38 MAPK (MAPK14) were included to create the protein-protein interaction network. Remarkably, network analysis revealed that Akt1 interacted both with NF-κB p65 and p38 MAPK, suggesting that Akt1 may play a role in role in the Numb-mediated regulatory network in macrophages (Fig. 6F). Although all three Akt isoforms (Akt1, Akt2, Akt3) share a high degree of homology, *in vitro* studies showed no difference in the ability of these isoforms to recognize and phosphorylate protein substrates^48^. We observed increased phosphorylation of Akt in LPS-stimulated macrophages wherein *Numb* was silenced (Fig. 6G); however, levels of *Akt2* in *Numb*-silenced macrophages and wild-type cells did not differ (Supplementary Figure 4). These results were consistent with studies of macrophages from *Akt1* knockout mice, in which TNFα and IL-6 were up-regulated^49^,^50^. Taken together, these data report a novel and as-yet-undescribed function for Numb, as a regulator of pro-inflammatory cytokines in macrophages through Numb-Itch interactions as well as through the Akt pathway which affects both p38 MAPK and NF-κB pathways, in addition to its role as a negative regulator of Notch signaling (Fig. 6H).

## Discussion

To our knowledge, the function of Numb in activated macrophage has never been documented. Here we describe both Notch-dependent and -independent roles for Numb in macrophage activation. As reported for other cell types, Numb was found to affect diverse signaling pathways in macrophages which might ultimately dictate the outcomes of the inflammatory responses. Our data showed that the amount of activated, intracellular Notch was higher in LPS-stimulated macrophages wherein *Numb* was silenced, suggesting that Numb functions as a negative regulator of Notch signaling. In addition, we also found that the Notch target, *Hes1*, was elevated following the activation in *Numb*-deficient macrophages. These results are consistent with other reports demonstrating that Numb is a negative regulator of Notch signaling in other cell types^45^,^51^. Therefore, we can extend this role for Numb as a negative regulator of Notch signaling to include macrophages.

Numb was first reported as a regulator of cell fate determinant by interrupting Notch signaling in sensory organ progenitors of *Drosophila melanogaster* and in mammalian retinal cells and neurons^26^,^30^,^52^,^53^. Previous studies using hematopoietic bone marrow cells, demonstrated that activating Notch signaling using immobilized Delta-1 resulted in apoptosis and inhibited monocyte into macrophage differentiation under M-CSF stimulation^54^. However, our results demonstrate that sustained Notch activity, as a result of interrupting Numb in bone marrow, did not affect monocyte differentiation into macrophages. Consistent with our results, studies using *Numb* and *Numb-like* double-knockout mice revealed that bone marrow cells, including macrophages, developed and distributed normally^16^. When Numb was overexpressed to inhibit Notch signaling in T cells, T cell maturation and development also remained intact^55^. These results imply that Numb may influence cellular differentiation in a context-dependent manner.

To our surprise, silencing *Numb* resulted in down-regulation of pro-inflammatory cytokines such as TNFα, IL-6, and IL-12p40, despite activation of Notch signaling. Furthermore, silencing *Numb* strongly affected the activation of p38 MAPK and NF-κB p65. Recently, Itch was demonstrated to be a negative regulator of NF-κB and p38 MAPK signaling pathways that control pro-inflammatory cytokines, mainly through ubiquitination and degradation of Tak1 and Tab1^46^,^56^,^57^. We found that Itch expression increased in LPS-activated, *Numb*-silenced macrophages, suggesting that Numb and Itch may counter-regulate signaling cascades to control the expression of pro-inflammatory cytokines in macrophages. Additionally, Itch has been identified as a Numb binding partner in order to regulate Notch signaling in other cell types^45^,^58^,^59^. To this end, we demonstrated that Numb interacts with Itch in resting macrophages and they dissociate after LPS stimulation. Therefore, upon LPS stimulation, Itch may be released from Numb to function as a negative regulator of pro-inflammatory response. Understanding the precise means by which Itch and Numb interact will require further investigation.

From our proteomics data, we constructed a network of proteins capable of regulating pro-inflammatory cytokines, in which Akt1 was located at the center and interacted with NF-κB p65 and p38 MAPK. In addition, quantitative proteomics and mRNA analyses revealed that the expression level of Akt1 increased in *Numb*-deficient macrophages. These results suggested that Akt1 also played a role in regulating pro-inflammatory cytokine production in macrophages. In support of our results, studies in *Akt1*^*−/−*^ mice showed that LPS stimulation of *Akt1*^*−/−*^ macrophages gave rise to pro-inflammatory phenotypes which included secretion of more TNFα and IL-6^49^,^50^. There are three Akt isoforms each of which displays distinct tissue distribution and function. Akt1 is required for induction of nitric oxide synthase and endothelial cell function whereas Akt2 is required for insulin-responsive signaling^60^,^61^,^62^. In contrast, Akt3 is involved in brain development and function; however, its function remains largely undefined^63^,^64^. We observed an upregulation of Akt1 in *Numb*-deficient macrophages, whereas *Akt2* remained comparable to that of wild-type. In addition, we showed that the phosphorylation level of Akt was higher in LPS-stimulated, *Numb*-deficient macrophages. Taken together, our results are consistent with a model showing Numb regulates multiple pathways as depicted in Fig. 6H. In this model Numb negatively regulates Notch, Akt1 and Itch, and each pathway further affects defined downstream signaling. Exactly how Numb regulates Akt levels is a subject for further investigation.

## Additional Information

**How to cite this article**: Kueanjinda, P. *et al.* A Novel Role of Numb as A Regulator of Pro-inflammatory Cytokine Production in Macrophages in Response to Toll-like Receptor 4. *Sci. Rep.* **5**, 12784; doi: 10.1038/srep12784 (2015).

## Supplementary Material

Supplementary Information
