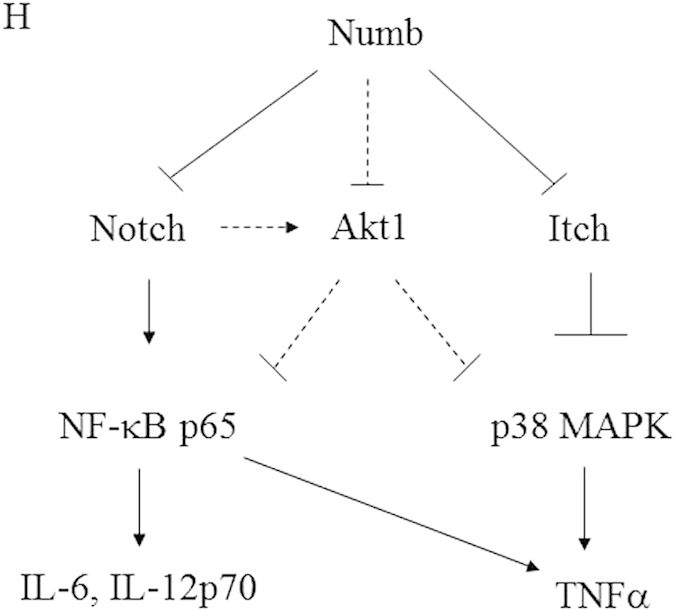# Corrigendum: A Novel Role of Numb as A Regulator of Pro-inflammatory Cytokine Production in Macrophages in Response to Toll-like Receptor 4

**DOI:** 10.1038/srep14585

**Published:** 2015-10-05

**Authors:** Patipark Kueanjinda, Sittiruk Roytrakul, Tanapat Palaga

Scientific Reports 5: Article number: 1278410.1038/srep12784; published online: 08052015; updated: 10052015

In this Article, Fig. 6H is a duplication of Supplementary Figure 4. The correct Fig. 6H appears below as Fig. 1.

## Figures and Tables

**Figure 1:**