# Supplementary material for: A Novel Role of Numb as A Regulator of Pro-inflammatory Cytokine Production in Macrophages in Response to Toll-like Receptor 4
Source: Sci Rep. 2015 Aug 5;5:12784. doi: 10.1038/srep12784 (PMC4542673; doi:10.1038/srep12784)
Supplement: Supplementary Information [file srep12784-s1.pdf]

A Novel Role of Numb as A Regulator of Pro-inflammatory Cytokine Production in Macrophages in Response to Toll-like Receptor 4

Patipark Kueanjinda, Sittiruk Roytrakul & Tanapat Palaga

Supplementary Information

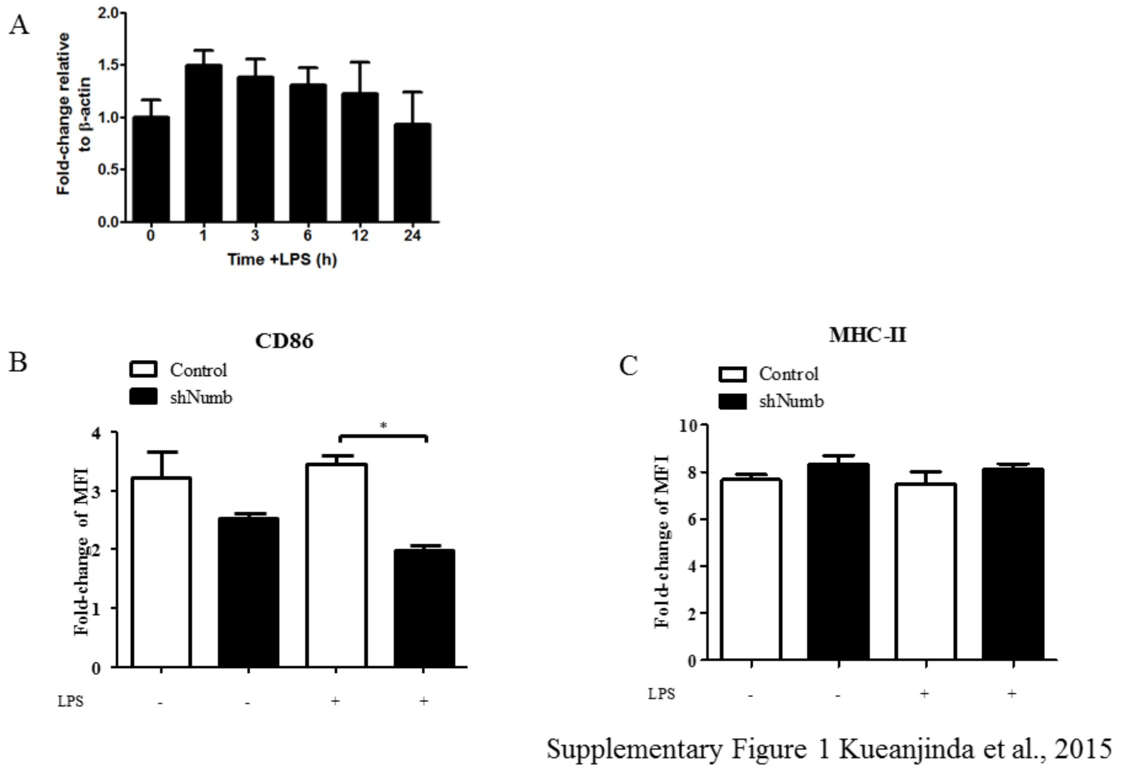

Supplementary Figure 1. Effect of Numb deletion on CD86 and MHCII in macrophages.

(A) Bone marrow-derived macrophages were stimulated with LPS (100 ng/mL) and assessed for Numb protein expression. Data are mean ± SEM of three independent experiments.

(B-C) The levels of CD86 (B) and MHC-II (C) expressed on macrophages from gated GFP$^+$ population of macrophages retrovirally infected with control (open bar) or sh*Numb* (closed bar) vectors were determined by flow cytometry. Data are the mean ± SEM calculated from fold-change of mean fluorescence intensity (MFI) from three independent experiments.

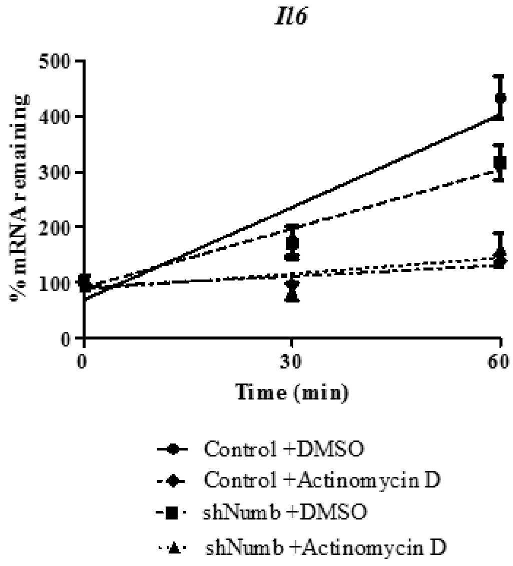

Supplementary Figure 2 Kueanjinda et al., 2015

Supplementary Figure 2. Stability of *Il6* mRNA is not affected by Numb deletion in macrophages. GFP+ macrophages containing pMKO.1-GFP or pMKO.1-shNumb-GFP were stimulated with LPS (100 ng/mL) for 1 hr prior to treatment with DMSO or actinomycin D (ActD) to inhibit RNA synthesis. The relative amount of remaining *IL-6* mRNA were measure by real-time PCR. Data are representative of two independent experiments.

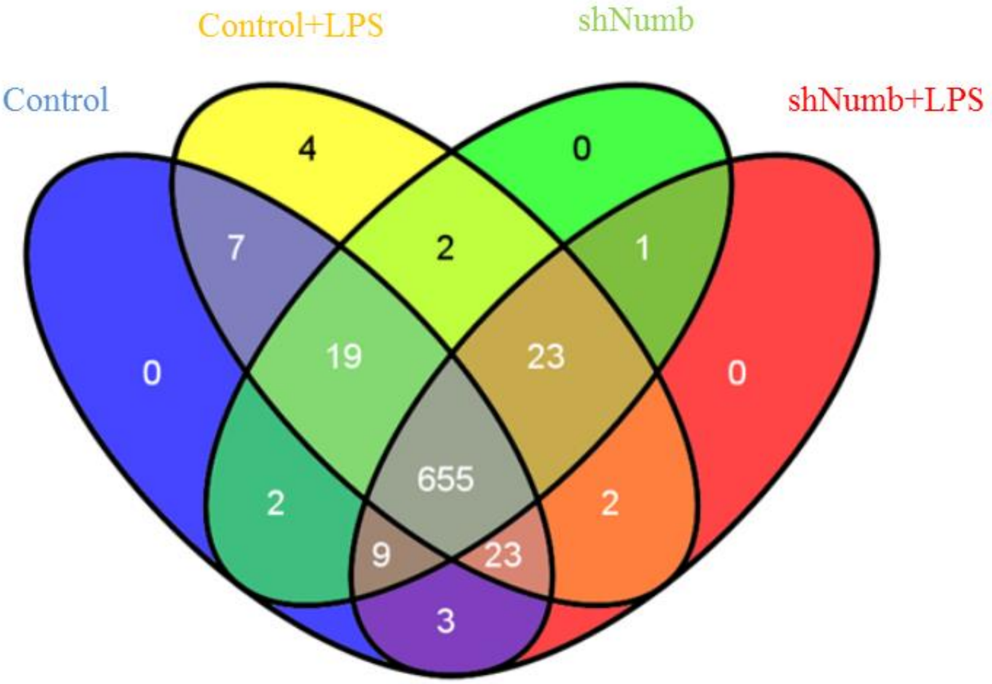

Supplementary Figure 3 Kueanjinda et al., 2015

Supplementary Figure 3. Numb suppression leads to changes of proteins in macrophages. Venn diagram displays the number of LPS-inducible proteins that were differentially changed in *Numb*-silenced macrophages. Macrophages retrovirally transduced with pMKO.1-GFP (Control) or pMKO.1-shNumb-GFP (shNumb) were treated with LPS for 30 min (Control+LPS and shNumb+LPS). The diagram was created using web-based Venny [1].

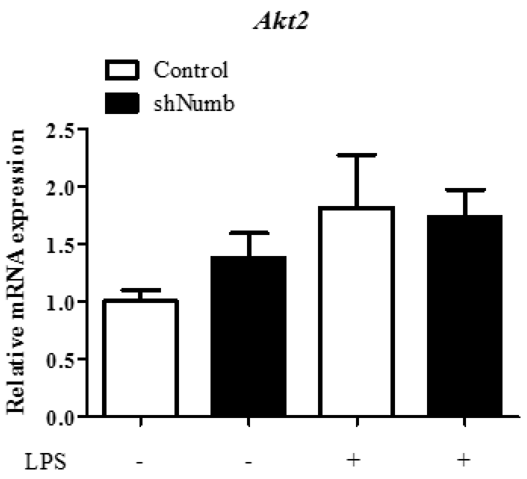

Supplementary Figure 4 Kueanjinda et al., 2015

Supplementary Figure 4. Expression of *Akt2* mRNA not affected by Numb silencing in macrophages. GFP^+ macrophages containing control or shNumb were stimulated with LPS (100 ng/mL) for 30 mins. The level of *Akt2* mRNA in control (open bars) or shNumb (closed bars) macrophages was detected by qPCR.

SUPPLEMENTARY MATERIALS

1. Pre-fractionation protein by SDS-PAGE and in-gel digestion

Proteins were fractionated on SDS-PAGE mini slab gel (8 x 9 x 0.1 cm, AE-6530 mPAGE, ATTO, Japan). The amount of protein for each sample was 15µg. Low molecular weight protein standard marker (Amersham Biosciences, UK) was used to estimate size of polypeptides. Electrophoresis was performed in SDS electrophoresis buffer (25mM Tris-HCl pH 8.3, 192mM glycine, 0.1% SDS), and the gels were silver stained according to Blum et al.[2]. After protein bands were excised into pieces, the gel plugs were dehydrated with 100% acetonitrile (ACN) and reduced with 10mM DTT. Afterward, the gel pieces were dehydrated twice with 100% ACN for 5 min. Then, add 10 µl of trypsin solution (10 ng/µl trypsin in 50% ACN/10mM ammonium bicarbonate) to the gels followed by incubation at room temperature for 20 min. Thereafter, add 20 µl of 30% to the gel and incubate at 37°C overnight. On the next day, 30 µl of 50% ACN in 0.1% formic acid was added to the gels to extract peptide digestion products. The gels were incubated at room temperature for 10 min in a shaker. Then, peptides extracted were collected and pooled together in the new tube. The pool extracted peptides were dried by vacuum centrifuge and kept at -80°C for further mass spectrometric analysis.

2. HCTUltra LC-MS analysis

Peptide solutions were analyzed using an HCTultra PTM Discovery System (Bruker Daltonics Ltd., U.K.) coupled to an UltiMate 3000 LC System (Dionex Ltd., U.K.). Peptides were separated on a nanocolumn (PepSwift monolithic column 100 µm i.d. x 50 mm). Peptide fragment mass spectra were acquired in data-dependent AutoMS (2) mode with a scan range of 300−1500 *m*/*z*, 3 averages, and up to 5 precursor ions selected from the MS scan 50−3000 *m*/*z*. Peptide peaks were detected and deconvoluted automatically using DataAnalysis version 4.0

(Bruker). Mass lists in the form of Mascot generic files were created automatically and used as the input for Mascot MS/MS Ions searches of the National Center for Biotechnology Information non-redundant (NCBI nr) database (www.matrixscience.com). Default search parameters used were the following: Enzyme = trypsin, max. missed cleavages =1; fixed modifications = carbamidomethyl (C); variable modifications = oxidation (M); peptide tolerance ±1.2 Da; MS/MS tolerance ±0.6 Da; peptide charge = 1+, 2+ and 3+; instrument = ESI-TRAP.

3. Proteins quantitation and identification

For proteins quantitation, DeCyder MS Differential Analysis software (DeCyderMS, GE Healthcare [3,4] was used. Acquired LC-MS raw data were converted and the PepDetect module was used for automated peptide detection, charge state assignments, and quantitation based on the peptide ions signal intensities in MS mode. The analyzed MS/MS data from DeCyderMS were submitted to database search using the Mascot software (Matrix Science, London, UK, [5]). The data was searched against the NCBI database for protein identification. Database interrogation was; taxonomy (*Mus musculus*); enzyme (trypsin); variable modifications (carbamidomethyl, oxidation of methionine residues); mass values (monoisotopic); protein mass (unrestricted); peptide mass tolerance (1 Da); fragment mass tolerance (±0.4 Da), peptide charge state (1+, 2+ and 3+) and max missed cleavages (1). Proteins considered as identified proteins had at least two peptides with an individual mascot score corresponding to $p<0.05$ and $p<0.1$, respectively.

REFERENCES

1 Oliveros, J. C. VENNY. An interactive tool for comapring lists with Venn Diagrams. (2007). http://bioinfogp.cnb.csic.es/tools/venny/index.html.

2 Blum, H., Beier, H. & Gross, H. J. Improved silver staining of plant proteins, RNA and DNA in polyacrylamide gels. *Electrophoresis* **8**, 93-99, doi:10.1002/epls.1150080203 (1987).

3 Johansson, C. *et al.* Differential expression analysis of Escherichia coli proteins using a novel software for relative quantitation of LC-MS/MS data. *Proteomics* **6**, 4475-4485, doi:10.1002/pmic.200500921 (2006).

4 Thorsell, A., Portelius, E., Blennow, K. & Westman-Brinkmalm, A. Evaluation of sample fractionation using micro-scale liquid-phase isoelectric focusing on mass spectrometric identification and quantitation of proteins in a SILAC experiment. *Rapid communications in mass spectrometry : RCM* **21**, 771-778, doi:10.1002/rcm.2898 (2007).

5 Perkins, D. N., Pappin, D. J., Creasy, D. M. & Cottrell, J. S. Probability-based protein identification by searching sequence databases using mass spectrometry data. *Electrophoresis* **20**, 3551-3567, doi:10.1002/(SICI)1522-2683(19991201)20:18<3551::AID-ELPS3551>3.0.CO;2-2 (1999).
